# Purtscher-like retinopathy in a patient with COVID-19-associated coagulopathy

**DOI:** 10.5935/0004-2749.2020-0328

**Published:** 2025-08-22

**Authors:** Joyce N Mbekeani, Nilesh K Raval, Thomas A Vo, Irene M Rusu, Sophie Zhirong Lin, Christine M Coyle, Julie P Hoffman

**Affiliations:** 1 Department of Surgery (Ophthalmology), Jacobi Medical Center, Bronx, NY; 2 Department of Ophthalmology & Visual Sciences, Montefiore Medical Center/Albert Einstein College of Medicine, Bronx, NY; 3 Brooklyn Eye Center, Brooklyn NY; 4 Department of Surgery, Montefiore Medical Center/Albert Einstein College of Medicine, Bronx, NY; 5 Department of Medicine (Infectious Diseases), Jacobi Medical Center, Bronx, NY; 6 Department of Medicine (Infectious Diseases), Montefiore Medical Center/Albert Einstein College of Medicine, Bronx, NY

**Keywords:** Retinal disease, Coronavirus infection, Severe acute respiratory syndrome, Case report, Doença retiniana, Infecção por coronavírus, Síndrome respiratória aguda grave, Relato de caso

## Abstract

The most frequently reported ophthalmic manifestation of severe acute respiratory syndrome coronavirus 2 (SARS-CoV-2) infection is conjunctivitis. We have described a case of Purtscher-like retinopathy in a patient with severe coronavirus disease 2019 (COVID-19)-associated coagulopathy. A young woman with multiple comorbidities was admitted for COVID-19-related acute respiratory distress syndrome. Her course was complicated by fungemia. Ophthalmic examination revealed bilateral posterior pole, intraretinal lesions and fluconazole was added for presumed fungal retinitis. At 1-week follow-up, widespread peripapillary cotton-wool spots and hemorrhages suggestive of Purtscher-like retinopathy were observed. The levels of D-dimers, fibrinogen, and C-reactive protein were markedly elevated prior to our consultation, indicating preceding prothrombotic and pro-inflammatory states. Subsequent venous duplex revealed deep venous thrombosis in the right subclavian and internal jugular veins. Von Willebrand factor indices were markedly elevated, suggesting severe COVID-19-associated coagulopathy. Purtscher-like retinopathy, a rare occlusive microangiopathy has been described in various pro-inflammatory and prothrombotic conditions. To the best of our knowledge, this is the first report of Purtscher-like retinopathy in COVID-19-associated coagulopathy.

## Abstract (pt)

A manifestação oftálmica mais frequentemente relatada da infecção por SARS-CoV-2 é a conjuntivite. Trata-se de estudo de caso de retinopatia tipo Purtscher em uma paciente com coagulopatia grave associada ao COVID-19. Uma jovem com múltiplas comorbidades foi admitida por síndrome do desconforto respiratório agudo relacionado ao COVID-19. Seu quadro foi complicado pela fungemia. O exame oftálmico revelou pólo posterior bilateral, lesões intraretinianas e o fluconazol foi adicionado para tratar a retinite fúngica presumida. No decorrer de uma semana, manchas largas peripapilares de algodão e hemorragias sugestivas de retinopatia tipo Purtscher foram observadas. Os dímeros D, o fibrinogênio e a proteína c-reativa estavam acentuadamente elevados antes da nossa consulta, indicando um estado pró-trombótico e pró-inflamatório precedente. O duplex venoso subsequente revelou trombose venosa profunda nas veias subclávia direita e jugular interna. Os índices de fatores von Willebrand estavam marcadamente elevados, sugerindo coagulopatia grave associada ao COVID-19. A retinopatia tipo Purtscher, uma microangiopatia oclusiva rara foi descrita em várias condições pró-inflamatórias e pró-trombóticas. Para nosso conhecimento, este é o primeiro relatório de retinopatia tipo Purtscher com coagulopatia associada ao COVID-19.

## INTRODUCTION

Purtscher retinopathy is a rare occlusive microangiopathy characterized by cotton-wool spots and intraretinal hemorrhages in the posterior pole. Purtscher flecken, small areas of inner retinal whitening between the arterioles and venules, occur in the acute phase^(1)^.

Originally described in association with trauma, similar retinal patterns have been reported in non-traumatic conditions, including pancreatitis, autoimmune disorders, and renal failure and they have been designated as Purtscher-like retinopathy (PLR). Although not fully understood, peripapillary capillary occlusion by endothelial damage and thrombosis or microembolism and the upregulation of the complement cascade have been implicated pathogeneses^(1)^. Typically, a clinical diagnosis, it takes hours to days following the inciting disorder to manifest the signs of peripapillary retinal cotton-wool spots, hemorrhages, and disc edema^(1)^. Herein, we have described a patient with coronavirus disease 2019 (COVID-19)-associated coagulopathy and retinal features suggestive of PLR. To the best of our knowledge, the present case is the first observation of this association.

## CASE REPORT

A 30-year-old woman, with a history of poorly controlled type II diabetes mellitus, hypertension, systemic lupus erythematosus (SLE)/rheumatoid arthritis (RA) over lapping syndrome, asthma, morbid obesity, and obstructive sleep apnea was admitted for coronavirus disease 2019 (COVID-19)-related pneumonia, as confirmed through nasopharyngeal swab reverse-transcriptase-polymerase chain-reaction (RT-PCR) assay for severe acute respiratory syndrome coronavirus 2 (SARS-CoV-2). At-home medications were continued including hydroxychloroquine for SLE/RA. The patient was then placed on azithromycin for COVID-19; however, she failed to meet the criteria for remdesivir trial therapy considering renal insufficiency. She was intubated for progressive respiratory distress and hypoxemia.

Her intensive care unit (ICU) course was complicated by septic shock and acute renal failure. Her blood cultures were positive for Staphylococcus similans and Candida glabrata, which were managed with vancomycin and caspofungin. Her right arm was noted to be swollen and her D-dimer levels had increased to 3,124 ng/mL (normal range: 0-230). COVID-19-associated coagulopathy (CAC) was suspected, and the anticoagulation regime was changed from prophylactic to weight-based dosing. Subsequent upper extremity venous duplex examination revealed right subclavian and internal jugular vein thromboses.

**Figure 1:**
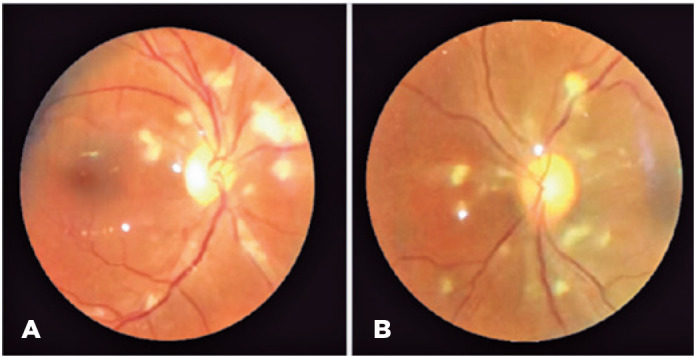
Images of the retina of a patient with COVID-19-associated coagulopathy showing bilateral posterior pole cotton-wool spots, segmental optic disc edema, and a small hemorrhage (in the right eye), suggestive of Purtscher-like retinopathy. Images were captured with 20 diopter lens and smartphone at an ICU patient bedside.

Ophthalmology was consulted to rule out ocular involvement from fungemia. The patient was asymptomatic, and her examination revealed bilateral uncorrected visual acuities of 20/25, round and reactive pupils without afferent pupillary defects, and grossly normal anterior segments. Dilated examination disclosed bilateral, white intraretinal posterior pole deposits without peripheral retina or vitreous involvement. Fluconazole was added for better ocular penetration for presumed fungal retinitis. After 1-week, dilated fundoscopy revealed bilateral segmental disc edema, and widespread posterior pole cotton-wool spots with a small hemorrhage in the right eye, which were suggestive of PLR (Figure 1).

**Figure 2:**
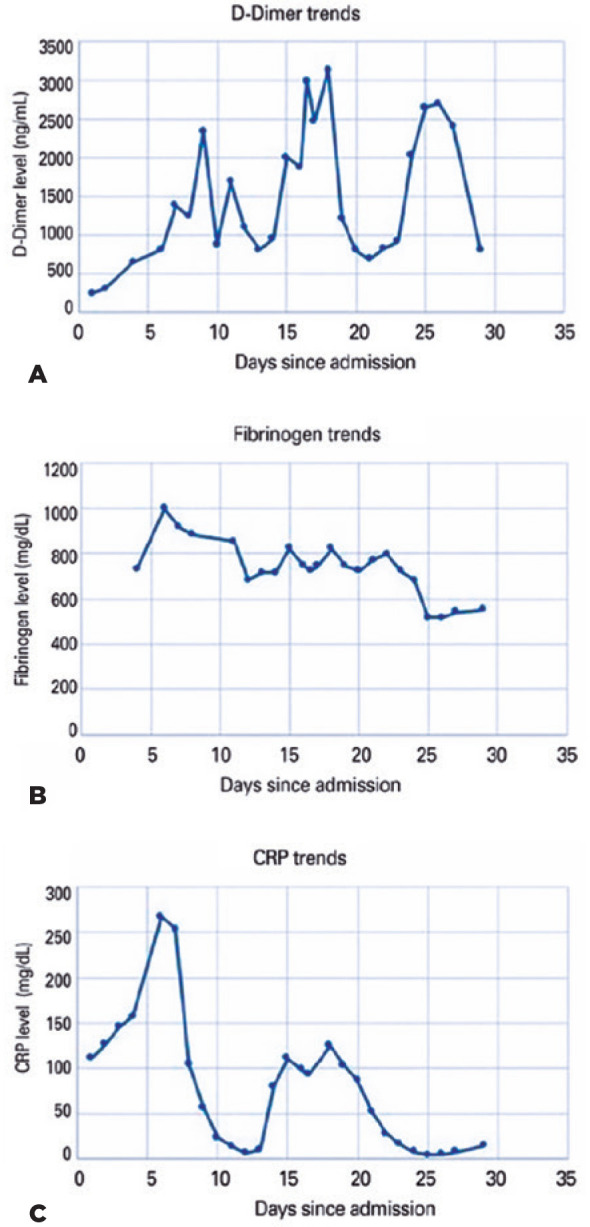
(A) D-dimer trends: mean= 1477 ng/mL, range= 255-3, 124 (normal= 0-230 ng/mL), peak: day 18; (B) Fibrinogen trends: mean= 736 mg/dL, range= 517 _≥_1000 (normal= 200400 mg/dL), peak: day 6; (C) C-reactive protein (CRP) trends: mean = 78 mg/L, range= 6.1-267.4 (normal= <10 mg/L), peak day: 6. Early intense inflammation and prothrombotic state were evidenced by the peak values of acute phase reactants, fibrinogen, and CRP. This was followed by coagulopathy with elevation of the levels of D-dimers. In this time period, platelets, prothrombin time, and activated partial thromboplastin time were within the normal limits.

Laboratory work-up revealed markedly elevated levels of D-dimers, fibrinogen, and C-reactive protein (Figure 2A-C). The prothrombin time, activated partial thromboplastin time (aPTT), and platelet count were within normal limits throughout the admission. The von Willebrand indices were abnormally elevated: factor VIII was 307 (normal= 50%-150%), von Willebrand antigen was 431 (normal= 40%-140%), and von Willebrand activity was 356 (normal= 40%-130%). Anti-cardiolipin and β-2 glycoprotein antibodies were within the normal limits. These findings suggested CAC. Further inpatient ophthalmic examinations revealed similar retinal lesions with minimal changes. The patient was discharged after 35 days of hospitalization on long-term anticoagulation therapy and fluconazole. Her follow-up was scheduled for repeat fundoscopic examination, optical coherence tomography, and fundus fluorescein angiography.

## DISCUSSION

COVID-19 disease, caused by SARS-CoV-2, has protean clinical manifestations with various pathogeneses. Angiotensin-converting enzyme-2 (ACE2) receptors, the adhesion site of SARS-CoV-2, plays a vital role in the pathogenesis of COVID-19 disease. These receptors are abundant in several tissues, including the alveolar and vascular endothelial cells^(2)^. Current evidence suggeststhat virus affinity for endothelial cell ACE2 receptors upregulates endothelial cell apoptosis and proinflammatory and prothrombotic cascades that may be respon sible for CAC^(3,4)^. A recent series provided support for this contention. Autopsy specimens from the lungs of COVID-19 patients who died with acute respiratory distress syndrome (ARDS) exhibited a 9-fold greater prevalence of alveolar capillary microthrombi than those with ARDS and influenza A (H1N1). Furthermore, transmission electron microscopy revealed extensive endothelial cell damage and intracellular and extracellular SARS-CoV-2^(4)^.

In our patient, coagulopathy was likely multifactorial, including the known prothrombotic comorbidities (i.e., diabetes mellitus, hypertension, morbid obesity, SLE/RA), sepsis, mechanical ventilation, renal failure, and prolonged immobility while in the ICU. Anti-phospholipid syndrome, often associated with SLE, was likely non-contributory in this instance since the levels of anti-cardiolipin and β-2 glycoprotein, were normal. However, CAC likely played a pivotal role. The elevated levels of D-dimers, an index of coagulation and fibrin degradation, fibrinogen (Figure 2), von Willebrand factor antigen and activity, and factor VIII, which have been observed in other cases of CAC, support this consideration^(3)^. Although both CAC and disseminated intravascular coagulation (DIC) exhibit markedly elevated D-dimers, patients with CAC do not display the consumptive component of DIC, and typically have normal platelet counts and elevated fibrinogen, clotting factor VIII levels, and von Willebrand factor indices. Abnormal von Willebrand factor kinetics is an indication of extensive vascular endothelial cell activation and damage^(3)^.

The PLR observed in our patient may have resulted from widespread coagulopathy, microembolism, direct local microvasculopathy from SARS-CoV-2 endotheliopathy, or a combination of these mechanisms. Intraretinal lesions observed on the initial examination were believed to be fungal deposits, but they may have been Purtscher flecken, the acute phase manifestations of PLR^(4)^. Recently, 4 patients with COVID-19 were reported to exhibit subtle cotton-wool spots and microhemorrhages^(5)^. Although the exact pathogenesis of these lesions is unknown, these retinal changes may be related to viral infection or represent form-fruste PLR.

SARS-CoV-2 has been detected in the cerebral spinal fluid^(6)^ and tears^(7)^ but is yet to be identified within the eye. Experimental animal studies suggest that other coronaviruses possess the capacity to cause anterior uveitis, choroiditis, retinal vasculopathies, and optic neuritis^(8,9)^. ACE2 receptors have been identified in various human ocular tissues, including the retina and retinal blood vessels^(2,10)^. While this virus has previously been described to cause conjunctivitis, it is plausible that ophthalmic manifestations of COVID-19 disease are diverse and operate through a variety of pathogeneses. Our appreciation of the full scope of clinical expressions of SARS-CoV-2 infection is still evolving and warrants future clinical and autopsy investigations to help elucidate the complete spectrum of ophthalmic manifestations.
